# Post-exercise branched chain amino acid supplementation does not affect recovery markers following three consecutive high intensity resistance training bouts compared to carbohydrate supplementation

**DOI:** 10.1186/s12970-016-0142-y

**Published:** 2016-07-26

**Authors:** Wesley C. Kephart, Petey W. Mumford, Anna E. McCloskey, A. Maleah Holland, Joshua J. Shake, C. Brooks Mobley, Adam E. Jagodinsky, Wendi H. Weimar, Gretchen D. Oliver, Kaelin C. Young, Jordan R. Moon, Michael D. Roberts

**Affiliations:** 1 School of Kinesiology, Molecular and Applied Sciences Laboratory, Auburn University, 301 Wire Road, Office 286, Auburn, AL 36849 USA; 2 Edward Via College of Osteopathic Medicine – Auburn Campus, Auburn, AL USA; 3 American Public University System, School of Health Sciences, Charles Town, WV USA

**Keywords:** Branched chain amino acids, Resistance training, Muscle damage, Immune system

## Abstract

**Background:**

Amino acid supplementation has been shown to potentially reduced exercise-induced muscle soreness. Thus, the purpose of this study was to examine if branched chain amino acid and carbohydrate (BCAACHO) versus carbohydrate-only sports drink (CHO) supplementation attenuated markers of muscle damage while preserving performance markers following 3 days of intense weight training.

**Methods:**

Healthy resistance-trained males (*n* = 30) performed preliminary testing (T1) whereby they: 1) donated a baseline blood draw, 2) performed knee extensor dynamometry to obtain peak quadriceps isometric and isokinetic torque as well as electromyography (EMG) activity at 60°/s and 120°/s, and 3) performed a one repetition maximum (1RM) barbell back squat. The following week participants performed 10 sets x 5 repetitions at 80 % of their 1RM barbell back squat for 3 consecutive days and 48 h following the third lifting bout participants returned for (T2) testing whereby they repeated the T1 battery. Immediately following and 24 h after the three lifting bouts, participants were randomly assigned to consume one of two commercial products in 600 mL of tap water: 1) BCAAs and CHO (3 g/d L-leucine, 1 g/d L-isoleucine and 2 g/d L-valine with 2 g of CHO; *n* = 15), or 2) 42 g of CHO only (*n* = 15). Additionally, venous blood was drawn 24 h following the first and second lifting bouts and 48 h following the third bout to assess serum myoglobin concentrations, and a visual analog scale was utilized prior, during, and after the 3-d protocol to measure subjective perceptions of muscular soreness.

**Results:**

There were similar decrements in 1RM squat strength and isokinetic peak torque measures in the BCAA-CHO and CHO groups. Serum myoglobin concentrations (*p* = 0.027) and perceived muscle soreness (*p* < 0.001) increased over the intervention regardless of supplementation. A group*time interaction was observed for monocyte percentages (*p* = 0.01) whereby BCAA-CHO supplementation attenuated increases in this variable over the duration of the protocol compared to CHO supplementation.

**Conclusion:**

BCAA-CHO supplementation did not reduce decrements in lower body strength or improve select markers of muscle damage/soreness compared to CHO supplementation over three consecutive days of intense lower-body training.

## Background

High-intensity resistance exercise elicits muscle damage and this can be linked to diminished performance [1–4]. Indeed, there have been reviews regarding the efficacy of a wide array of nutritional ergogenic aids in optimizing strength and power [5, 6]. Specifically, supplemental protein (e.g. whey, casein, soy, and egg) and/or amino acid ingestion enhances post-exercise recovery by increasing skeletal muscle protein synthesis and reducing muscle proteolysis [7–9] while also potentially reducing post-exercise skeletal muscle inflammation and oxidative stress [10].

Researchers have also examined if amino acid supplementation can mitigate post-exercise muscle damage. Jackman et al. [11] reported that branched chain amino acid (BCAA) supplementation attenuated perceived muscle soreness in an untrained population, but supplementation did not alter muscle function following unilateral eccentric knee extension exercise. Howatson et al. [12] also reported that 7 days of BCAA ‘loading’ in resistance-trained participants attenuated increases in serum creatine kinase (a muscle damage marker) and perceived soreness following one bout of drop-jump induced muscle damage. Other research has shown that BCAAs may foster a beneficial hormonal environment as well as blunt the rise in serum creatine kinase levels following intense resistance exercise [13]. Nosaka et al. [14] reported that BCAA supplementation in untrained individuals reduced muscle damage and soreness when consumed immediately before and during a 4-d recovery period of an eccentric leg extensor bout. However, opposite findings also exist. For instance, White et al. [15] reported that carbohydrate/protein supplementation did not have an effect on muscle soreness, performance or serum creatine kinase levels following eccentric quadriceps contractions. Stock et al. [16] similarly reported that a leucine/carbohydrate beverage provided to subjects prior to and after 6 sets of heavy back squats did not affect serum creatine kinase, serum lactate dehydrogenase, or muscle soreness up to 72 h following exercise. These authors also reported that back squat performance was not improved with leucine/carbohydrate beverage administration 72 h following the initial heavy squat.

While there is some evidence to suggest that amino acid supplementation may be beneficial for promoting various facets of post-exercise skeletal muscle recovery (i.e., increasing muscle protein synthesis and reducing muscle protein breakdown), there are also studies finding that BCAAs are not useful in improving markers of muscle damage, soreness and performance during or following acute exercise protocols. Therefore, the purpose of this study was to compare the effects of BCAA and carbohydrate (BCAA-CHO) versus carbohydrate (i.e., sports drink; CHO) supplementation on markers of muscle damage and performance as well as gross immunological markers during and after a 3-day high intensity back squat protocol. Based upon the aforementioned literature, we hypothesized that BCAA-CHO would attenuate performance decrements and/or positively affect muscle damage markers compared to CHO supplementation.

## Methods

### Participant characteristics

Prior to initiating this study, the protocol was reviewed and approved by the Auburn University Institutional Review Ethics Committee (protocol #14-328), and was in compliance with the Helsinki Declaration. Apparently healthy males (*n* = 30) volunteered to take part in this investigation. Subjects gave written consent and completed the Physical Activity Readiness Questionnaire as well as a health history questionnaire to detect potential risk factors that might be aggravated by strenuous physical activity. All participants were resistance-trained, participating in ≥3 days per week of resistance exercise for at least 3 months*.*

### Experimental protocol

**Fig. 1:**
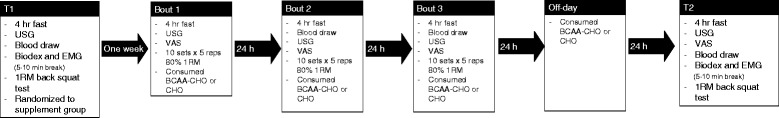
Study design. Abbreviations: USG, urine specific gravity; VAS, visual analog scale; EMG, electromyography; 1RM, one repetition maximum

Figure 1 provides an outline of the experimental protocol. The protocol is described more in-depth below and the experimental procedures used in the protocol are described thereafter.

#### Day 1 (T1)

Participants reported to the laboratory following a 4 h fast. In addition, participants were asked to forgo any strenuous activity for at least 48 h prior to arrival in order to minimize any residual markers of muscle damage. To assure adequate hydration status, urine specific gravity (USG) was measured using handheld refractometer (ATAGO 2393, Bellevue, WA, USA). The hydration threshold prior to exercise testing included a USG of 1.020 g•mL^-1^. For subjects that were dehydrated, 0.5 L of water were provided and USG was re-determined 30 min later. Baseline venous blood samples were then collected into a 5 mL serum separator tube and a 3 mL EDTA tube (BD Vacutainer, Franklin Lakes, NJ, USA) for subsequent analysis of serum and whole blood markers, respectively. Following blood draws, all participants consumed a cereal bar (Kellogg’s Nutri-Grain® bar; 2 g protein, 24 g carbohydrates, 3 g fat, 120 kcal) in order to standardize pre-testing meals. Participants then had their right mid-thigh shaved and alcohol-swabbed for electromyography (EMG) electrode placement and were seated on System 4 Pro Biodex isokinetic dynamometer (BioDex Medical Inc., New York, USA). Bottom start-position knee flexion was set at 90° and full knee extension was set at 10°. Following a familiarizing practice trial, participants performed a maximal knee extensor isometric contraction for 5 s in order to obtain peak torque and EMG maximal voluntary isometric contraction (MVIC) values. Following a brief recovery period (~1 min), maximal knee extensor isokinetic torque with EMG activity was measured across 5 repetitions at 60°/s and 120°/s, with a brief rest period (~1-2 min) between bouts.

A 5–10 min recovery period was allowed following isometric/isokinetic dynamometry. Following this recovery period, participants began a one repetition maximum (1RM) back squat protocol. The first set of back squats occurred with the bar only (20 kg). Participants were allowed a 2–3 min recovery period and then performed 5 repetitions at ~50 % of their putative 1RM. Participants were allowed a 2–3 min recovery period and then performed 2 repetitions at ~80 % of their putative 1RM. Thereafter, participants performed 1 rep whereby ~2–5 kg were added until they were unable to achieve a successful lift. To ensure that proper depth on each back squat repetition was accomplished, participants were asked to make contact with a box that was behind them without sitting on it entirely. The box was set at a height where the participant’s femur was perpendicular to the ground during the bottom-eccentric portion of the back squat. Following 1RM back squat testing, participants were scheduled subsequent experimental visits. In addition, they were asked to not perform any strenuous physical activity for at least 48 h prior to their day 2 visit described below.

#### Day 2 (bout 1)

Approximately one week following day 1 (T1), participants reported to the laboratory 4 h fasted, were checked for hydration status and were given a standardized cereal bar to standardize pre-exercise meals. Participants were then asked to mark on a visual analog scale to indicate their perceived soreness (described below). Participants then performed a warm up protocol of 5 repetitions at 20 %, 40 %, and 60 % of their back squat 1RM with 2–3 min between sets. Following the warm-up protocol, participants performed 10 sets of 5 repetitions at 80 % of their back squat 1RM with a 2–3 min recovery period between each set. If participants were unable to complete the repetitions, a 5 % reduction in resistance weight was employed. Lifting volumes for the bout 1 session were recorded and included dropped weights. Immediately following completion of this protocol, participants were randomly assigned to consume one of two commercial products in 600 mL of tap water and the products were provided to participants by laboratory testers. The composition of each product is described in further detail below:

1. BCAAs and CHO (BCAA-CHO) (2 servings of AMINO1, Musclepharm Corp., Denver, CO, USA); per 2 servings: 10 kcal, 3 g L-leucine, 1 g L-isoleucine and 2 g L-valine with 2 g of non-sugar carbohydrates
2. 42 g of carbohydrates (CHO; Powerade, Atlanta, GA, USA); per serving: 168 kcal, 40 g sugar whereby 57 % of the sugar is fructose, 39 % is glucose, 4 % is sucrose and the remainder of the sugars are trace amounts of lactose and maltose

While these supplements were not standardized to CHO or Calorie content, our intent was to provide a practical comparison between these two products. Alternatively stated, we surmised that (in a real-world setting) participants would either consume a sports drink-like CHO beverage or the experimental BCAA-CHO beverage and, thus, we compared two servings of each supplement regardless of Calorie or CHO content. Finally, we elected to provide participants two servings of BCAA-CHO given that two servings contain a total of 3 g of L-leucine and this amount has been posited to be a ‘threshold’ at which post-exercise muscle protein synthesis is optimized [17]. Of note, the testers and participants were blinded to the supplement conditions whereby BCAA-CHO were packaged in ‘B’ containers, and CHO was packaged in ‘A’ containers by a person in the laboratory who did not interact with persons involved in the study.

#### Day 3 (bout 2)

Twenty four hours following day 2 (bout 1), participants reported to the laboratory 4 h fasted, were checked for hydration status and blood was drawn/collected into a 5 ml serum tube and 3 ml EDTA tubes. Participants were then asked to mark on a visual analog scale to indicate their perceived soreness. Participants then performed the 10 sets of 5 repetitions at 80 % of their back squat 1RM described during day 2 above. Immediately following this exercise bout, participants were administered the same amount of BCAA-CHO or CHO that they had consumed on day 2 described above.

#### Day 4 (bout 3)

Twenty four hours following day 3 (bout 2), participants reported to the laboratory 4 h fasted, were checked for hydration status and blood was drawn/collected into a 5 ml serum tube and 3 ml EDTA. Again, participants were asked to mark on a visual analog scale to indicate their perceived soreness, and then perform the 10 sets of 5 repetitions at 80 % of their back squat 1RM described during days 2 and 3 above. Immediately following this exercise bout, participants were administered the same amount of BCAA-CHO or CHO that they had consumed on days 2 and 3 described above.

#### Day 5 (T2; 48 h following bout 3)

The T2 post-test occurred 48 h following day 4 (bout 3) described above, and the post-testing procedure was identical to the T1 described above. Of note, there was one full day of recovery between bout 3 and T2. While participants did not perform exercise during this recovery day, they were sent home with their respective supplement and were instructed to consume either the BCAA-CHO or CHO in order to further facilitate post-training recovery.

#### Other notes

Participants were asked to maintain their habitual dietary habits, and to ensure there were no potential between-group nutritional confounders, a four day food log was used to assess caloric and macronutrient intake. Food logs were analyzed for daily macronutrient and Caloric intake values using a free online resource [18]. Moreover, participants reported to the laboratory for testing or resistance training during the same time of day (±2 h).

### Experimental procedures

#### Visual analog scale

An adapted visual analog scale (VAS) was utilized to assess perceived muscular soreness as described previously [19]. Briefly, the scale was a straight line, 100 mm in length, and the participants were asked to “*mark on line below indicating how sore you are at this moment*”. The researcher explained that the most left aspect indicated no soreness at all, whereas the most right aspect indicates the most soreness that the participant has ever experienced.

#### EMG procedures

Bipolar Ambu BlueSensor M (Ambu INC, Columbia, MD, USA) surface electrodes (Ag/AgCl) were placed over the muscle belly of the right vastus lateralis (inter-electrode distance 25 mm), parallel to the muscle fibers using techniques described by Basmajian and Deluca [20]. Prior to electrode placement the participants’ mid-thighs were shaved, abraded and cleaned using alcohol swabs. A Noraxon Myosystem 1200 (Noraxon USA INC, Scottsdale, AZ) EMG system was used to obtain measurements of neuromuscular activation during MVIC and isokinetic trials. Surface EMG data were sampled at 1000 Hz. Raw EMG signals were full-wave rectified and filtered using a moving average with a 200 ms window. The MVIC was obtained during the isometric knee torque test. Peak values from MVIC trials were used to normalize peak values obtained during the isokinetic trials. Normalized isokinetic values were subsequently represented as percentage of MVIC.

#### Whole blood and serum analyses

On the days of blood collection, all 3 mL EDTA tubes were refrigerated at 4 °C. Following all testing for the day, tubes were transported to the CLIA-certified Auburn University Medical Clinic, and complete blood count (CBC) panels were analyzed using Beckman-Coulter DxH 600 Hematology analyzer (Beckman Coulter, Fullerton, CA, USA). Specifically, the following whole blood parameters were determined: total white blood cells (WBCs), neutrophil differentials (absolute counts and percentage of WBCs), lymphocyte differentials (absolute counts and percentage of WBCs), and monocyte differentials (absolute counts and percentage of WBCs).

On the days of blood collection, serum was also obtained from 5 ml serum collection tubes through centrifugation at 3500 x g for 5 min at room temperature. Serum aliquots were then placed in 1.7 ml microcentrifuge tubes and stored at -20 °C until batch-processing for serum myoglobin. A human ELISA for myoglobin was used to determine serum concentrations (Abcam, Cambridge, MA, USA). Of note, it has been shown that increases in serum myoglobin concentration is a valid marker of muscle-damage as well as being more sensitive and less variable than creatine kinase [21].

### Statistics

Unless otherwise stated, all variables are presented in figures and tables as means ± standard error values. Unless stated below, an alpha (α) level of *p* ≤ 0.05 was used to detect between- or within-group differences, and all statistics were performed using SPSS v22.0 (Chicago, IL, USA).

Independent t-tests were performed for pre-study training age, height, weight, BMI, and average daily dietary intakes between groups.

T1 and T2 1RM squat, isometric knee extensor torque, isokinetic knee extensor torque, and isokinetic knee extensor EMG activity were analyzed using 2x2 (group*time) mixed factorial ANOVAs. If a significant time α-value was observed for a dependent variable, subsequent paired samples t-tests were performed within each group. Moreover, given that only one comparison was performed within each group for each dependent variable (i.e., T1 vs. T2), raw p-values were used and Bonferroni adjustments were not applied. If a significant group*time α-value was observed for a dependent variable, subsequent paired sample t-tests were performed as described above and independent sample t-tests were also performed to locate specific differences between groups, respectively. Again, given that only one comparison was performed within or between each group for each dependent variable (i.e., T1 vs. T2), raw p-values were used and Bonferroni adjustments were not applied.

Total lifting volume was analyzed using 2x3 (group*time) mixed factorial ANOVAs and VAS data, and serum myoglobin concentrations and gross immunological variables were analyzed using 2x4 (group*time) mixed factorial ANOVAs. If a significant time α-value was observed for a dependent variable, subsequent pairwise comparisons with Bonferroni adjustments were applied within each group. If a significant group*time α-value was observed for a dependent variable, independent t-tests at each time point with manual Bonferroni adjustments were performed at each time point; thus, for the latter independent samples t-tests regarding VAS and serum data, *p* < 0.0125 was considered to be significant given that four comparisons were being made and manual Bonferroni adjustments were applied to these data.

It should be finally noted that, for all dependent variables analyzed with 2x2/3/4 mixed factorial ANOVAs, Mauchly’s tests of sphericity were performed to assure that the variances of all groups are equal and that the data were normally distributed. In the event that sphericity was not met, the Huynh-Feldt correction was applied to hypothesis testing.

## Results

### Participant characteristics

Participant characteristics are presented in Table 1. There were no between-group differences in height (*p* = 0.29), weight (*p* = 0.28), body mass index (*p* = 0.07), and age (*p* = 0.31). In addition, no between-group differences were observed for kcal/d intakes (*p* = 0.59), protein/d intakes (*p* = 0.70), carbohydrate/d intakes (*p* = 0.44), or fat/d intakes (*p* = 0.90) during the one-week intervention.

**Table 1:** Participant demographics and dietary intakes

Group	*n*	Age (yrs)	Height (cm)	Weight (kg)	BMI (kg/m^2^)	Caloric intake (kcal/d)	Protein (g/d)	Carbohydrates (g/d)	Fat (g/d)
BCAA-CHO	15	21.8 ± 0.4	180 ± 2	86.0 ± 3.5	26.6 ± 0.9	2697 ± 206	131 ± 12	303 ± 24	106 ± 11
CHO	15	22.5 ± 0.5	177 ± 2	87.7 ± 2.9	28.0 ± 0.8	2514 ± 207	125 ± 7	267 ± 29	104 ± 11

*Abbreviations*: *BCAA-CHO* branched-chain amino acid and carbohydrate-supplemented group, *CHO* carbohydrate only-supplemented group

No statistical differences were observed for any descriptive variables

### BCAA-CHO or CHO supplementation does not reduce decrements in 1RM squat strength and isometric knee extensor torque following three high-volume lifting bouts

**Fig. 2:**
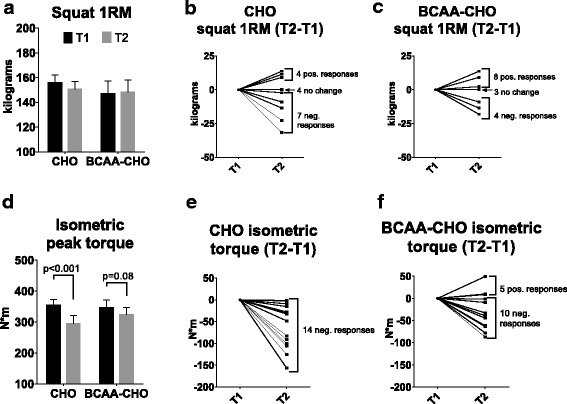
T1 and T2 squat and isometric peak torque values. No main time effect or group*time interaction was observed for 1RM squat (panel **a**). A main time effect was observed for isometric peak torque (panel **d**), and dependent *t*-tests revealed a significant decrease in the variable in the CHO group (*p* ≤ 0.001), while there was no decrease in the BCAA-CHO group (*p* = 0.08). Individual responses are plotted for squats (panel **b** and **c**) and isometric torque (panel **e** and **f**). Abbreviations: BCAA-CHO, branched-chain amino acid and carbohydrate-supplemented group; CHO, carbohydrate only-supplemented group

1RM squat performance failed to reach a significant main effect of time (*p* = 0.40) or a group*time interaction (*p* = 0.16; Fig. 2a; individual response graphs for 1RM squat are presented in Fig. 2b & c). Regarding peak isometric torque, a main effect of time was observed (*p* = 0.003) as both groups experienced decrements in this variable, and a group*time interaction approached significance (*p* = 0.06) although there were no between-group differences in this variable (Fig. 2d; individual response graphs for isometric peak torque are presented in Fig. 2e & f).

### Isokinetic torque or EMG variables were not differentially altered by BCAA-CHO or CHO supplementation

**Fig. 3:**
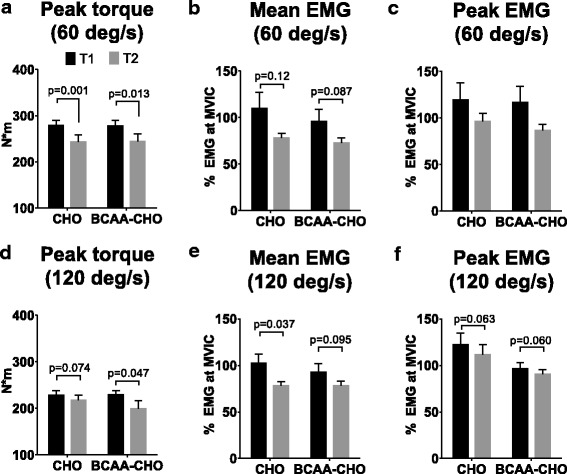
T1 and T2 isokinetic torque and EMG activity. A main time effect was observed for peak torque at 60°/s (*p* ≤ 0.001), and further analysis revealed T1-to-T2 decreases in the CHO (*p* = 0.001) and BCAA-CHO (*p* = 0.013) groups (panel **a**). A main time effect was observed for isokinetic mean EMG activity at 60°/s (*p* = 0.025), but no significant differences were observed within the CHO (*p* = 0.12) or BCAA-CHO (*p* = 0.087) groups (panel **b**). No main time effect or group*time interaction was observed for isokinetic peak EMG at 60°/s (panel **c**). There was a main time effect for isokinetic peak torque at 120°/s (*p* = 0.021), but further analysis revealed that this variable did not decrease in the CHO (*p* = 0.074) and did decrease in the BCAA-CHO (*p* = 0.047) group (panel **d**). There was a main time effect for isokinetic mean EMG 120°/s (*p* = 0.007), and further analysis revealed T1-to-T2 decreases in the CHO (*p* = 0.037) but not BCAA-CHO (*p* = 0.095) group (panel **e**). There was a main time effect for isokinetic peak EMG at 120°/s (*p* = 0.007), but further analysis revealed T1-to-T2 decreases did not occur in the CHO (*p* = 0.063) or BCAA-CHO (*p* = 0.060) groups (panel **f**). Abbreviations: BCAA-CHO, branched-chain amino acid and carbohydrate-supplemented group; CHO, carbohydrate only-supplemented group

Regarding isokinetic torque at 60^o^/s, a main effect of time occurred as both groups experienced decrements in this variable (*p* < 0.001), but there was no group*time interaction (*p* = 0.94; Fig. 3a). There was a main time effect for mean EMG activity at 60 ^o^/s (*p* = 0.025) as both groups experienced decrements in this variable, but there was no group*time interaction (*p* = 0.70; Fig. 3b). There was a main time effect for peak EMG activity at 60 ^o^/s (*p* = 0.037) as both groups experienced decrements in this variable, but there was no group*time interaction (*p* = 0.63; Fig. 3c).

Regarding isokinetic torque at 120^o^/s there was a main effect of time (*p* = 0.021) as both groups experienced decrements in this variable, but there was no group*time interaction (*p* = 0.17; Fig. 3d). There was a main time effect for mean EMG activity at 120 ^o^/s (*p* = 0.007) as both groups experienced decrements in this variable, but there was no group*time interaction (*p* = 0.45; Fig. 3e). There was a main time effect for peak EMG activity at 120 ^o^/s (*p* = 0.007) as both groups experienced decrements in this variable, but there was no group*time interaction (*p* = 0.75; Fig. 3f).

### BCAA-CHO or CHO supplementation does not differentially effect myoglobin levels, perceived muscle soreness, or training volume during or after three high intensity lifting bouts

**Fig. 4:**
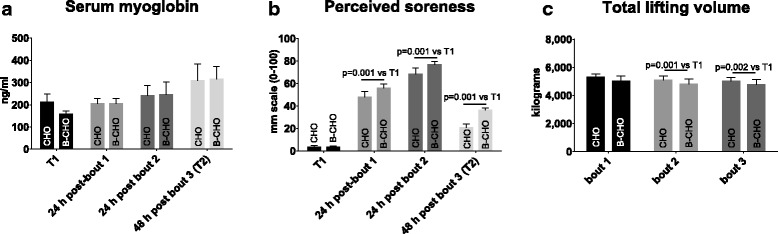
Serum myoglobin, perceived soreness, and training volume throughout the study. A main time effect was observed for myoglobin (*p* = 0.027), but pairwise comparisons (both groups collapsed over time) only revealed a tendency for this value to increase at T2 relative to T1 (*p* = 0.055) (panel **a**). A main time effect was observed for perceived soreness (*p* ≤ 0.001), and pairwise comparisons revealed increases 24 h post-bout 1 (*p* ≤ 0.001), 24 h post bout 2 (*p* ≤ 0.001), and 48 h post bout 3 (*p* ≤ 0.001) relative to T1 (panel **b**). There was a main time effect for total lifting volume (*p* ≤ 0.001), and pairwise comparisons revealed decreases at bout 2 (*p* ≤ 0.001) and bout 3 (*p* ≤ 0.001) relative to bout 1 (panel **c**). Abbreviations: BCAA-CHO, branched-chain amino acid and carbohydrate-supplemented group; CHO, carbohydrate only-supplemented group

Regarding serum myoglobin, there was a main effect of time (*p* = 0.027), but there was no group*time interaction (*p* = 0.87; Fig. 4a); specifically, pairwise comparisons (both groups collapsed over time) revealed a tendency for this value to increase at T2 relative to T1 (*p* = 0.055). There was a main effect of time for perceived muscle soreness (*p* < 0.001), but there was no group*time interaction (*p* = 0.17; Fig. 4b); specifically, pairwise comparisons revealed increases 24 h post-bout 1 (*p* ≤ 0.001), 24 h post bout 2 (*p* ≤ 0.001), and 48 h post bout 3 (*p* ≤ 0.001) relative to T1. Regarding resistance training volume on each of the exercise days, there was a main effect of time (*p* ≤ 0.001), but there was no group*time interaction (*p* = 0.92; Fig. 4c); specifically, pairwise comparisons revealed decreases at bout 2 (*p* ≤ 0.001) and bout 3 (*p* ≤ 0.001) relative to bout 1.

### Effects of BCAA-CHO versus CHO supplementation on whole blood variables

Whole blood variables over the duration of the study are presented in Table 2. There was no main effect for time (*p* = 0.37) or a group*time interaction (*p* = 0.15) regarding WBC counts. There was no main effect for time (*p* = 0.95) or a group*time interaction (*p* = 0.46) for neutrophil percentages. There was no main effect for time (*p* = 0.84) or a group*time interaction (*p* = 0.40) for neutrophil counts. There was no main effect for time (*p* = 0.42) or a group*time interaction (*p* = 0.69) for lymphocyte percentages. There was no main effect for time (*p* = 0.43) or a group*time interaction (*p* = 0.47) for lymphocyte counts. There was no main effect for time for monocyte percentages (*p* = 0.12), but a group*time interaction was observed (*p* = 0.01); specifically BCAA-CHO supplementation attenuated increases in this marker 24 h following bout 1 (*p* = 0.049), 24 h following bout 2 (*p* < 0.001), and 48 h following bout 3 (or at T2; *p* < 0.001) compared to CHO supplementation. However, analysis of monocyte counts failed to yield a significant effect of time (*p* = 0.29) or a group*time interaction (*p* = 0.55).

**Table 2:** Select white blood cell differentials between groups over the training intervention

Group	Bout	WBC (10^3^/μL)	Neutrophil Percent	Neutrophil Count (10^3^/μL)	Lymphocyte Percent	Lymphocyte Count (10^3^/μL)	Monocyte Percent	Monocyte Count (10^3^/μL)
BCAA-CHO	T1	6.51 ± 0.35	55.46 ± 2.45	3.64 ± 0.28	34.06 ± 2.41	2.03 ± 0.13	7.18 ± 0.32	0.49 ± 0.04
	24 h post bout 1	6.19 ± 0.26	56.77 ± 1.96	3.52 ± 0.21	31.79 ± 1.38	2.04 ± 0.15	7.48 ± 0.33	0.47 ± 0.03
	24 h post bout 2	6.43 ± 0.26	58.35 ± 2.51	3.97 ± 0.31	32.11 ± 2.33	1.89 ± 0.19	6.86 ± 0.31	0.44 ± 0.03
	48 h post bout 3 (T2)	6.56 ± 0.37	57.26 ± 1.81	3.69 ± 0.29	31.21 ± 1.90	1.98 ± 0.10	7.25 ± 0.24	0.49 ± 0.03
CHO	T1	6.44 ± 0.35	55.43 ± 2.59	3.53 ± 0.29	32.61 ± 2.03	2.11 ± 0.12	8.21 ± 0.45	0.57 ± 0.03
	24 h post bout 1	5.85 ± 0.32	53.54 ± 2.24	3.20 ± 0.28	32.43 ± 2.35	1.91 ± 0.12	**8.75 ± 0.52** ^**‡**^	0.50 ± 0.03
	24 h post bout 2	5.81 ± 0.33	53.18 ± 2.92	3.05 ± 0.28	33.76 ± 2.79	1.90 ± 0.13	**9.16 ± 0.40** ^**‡**^	0.55 ± 0.03
	48 h post bout 3 (T2)	6.21 ± 0.32	53.15 ± 2.06	3.34 ± 0.24	30.37 ± 2.14	1.90 ± 0.12	**9.15 ± 0.38** ^**‡†**^	0.59 ± 0.03

*Abbreviations BCAA-CHO* branched-chain amino acid and carbohydrate-supplemented group, *CHO* carbohydrate only-supplemented group

Analysis of monocyte percentages revealed a group*time interaction (*p* = 0.01) whereby BCAA-CHO supplementation attenuated increases in monocyte percentages 24 h following bout 1 (*p* = 0.049), 24 h following bout 2 (*p* < 0.001), and 48 h following bout 3 (or at T2; *p* < 0.001) compared to CHO supplementation (denoted by bold lettering and superscript ‡ symbol). Moreover, T2 monocyte percentages increased within the CHO group at T2 compared to T1 (denoted by superscript † symbol, *p* = 0.012)

## Discussion

Past studies are mixed regarding the effectiveness of BCAAs to mitigate muscle damage. Herein, we report that compared to CHO supplementation, BCAA-CHO supplementation was not able to reduce decrements in performance variables, markers of muscle damage and perceived muscle soreness increased over the study duration in both groups. These findings are discussed in greater detail below.

### BCAA-CHO supplementation does not reduce decrements in lower body strength after three consecutive high intensity exercise bouts

As mentioned previously, some reports suggest that BCAA supplementation reduces muscle soreness following muscle-damaging protocols, although they do not appear to aid in attenuating the reduction of muscular performance following intense resistance training [11, 12]. Our findings are in agreement with these reports in that BCAA-CHO-supplemented participants did not experience any significant performance outcomes relative to CHO-supplemented participants following three rigorous resistance exercise bouts. This contrasts the findings of Kirby et al. [22] who supplemented 250 mg/kg of leucine following 100 depth jumps and 6 sets of 10 repetitions on eccentric leg presses, and subsequently observed a greater maintenance of isometric squat force relative to placebo and control groups. The reason for our findings are likely related to the resistance training protocol being too much of a muscle-damaging stimulus. Alternatively stated, while limited evidence suggests that BCAA administration may prevent muscle soreness and markers of muscle damage [12], muscle damage inflicted by resistance exercise is likely not mitigated by nutritional factors and, thus, occurs in spite of macronutrient provision. Therefore, while BCAA provision following exercise enhances post-exercise muscle protein synthesis [23–26] and reduce muscle proteolysis [27–29], our findings suggest that BCAA-CHO supplementation does not prevent muscle fibers from being damaged during rigorous resistance training stimulus, and this ultimately leads to the performance decrements observed herein.

### BCAAs and CHO provision do not differentially alter myoglobin serum concentrations or muscle soreness

BCAAs have been reported to attenuate the rise in serum myoglobin [30] as well as attenuate perceived muscle soreness following rigorous exercise [12, 30]. However, our findings differ from the aforementioned reports. Reasons for the divergence of our findings could be due to differences in exercise protocols, as Shimomura et al. had participants perform one bout of 7 sets of 20 body-weighted back squat repetitions, whereas our investigation applied 3 consecutive days of high intensity heavy-weighted back squats. Further, Shimomura et al. administered a BCAA supplement prior to the exercise protocol, whereas we administered the supplement following exercise. Notwithstanding, our data are in agreement with White et al. [15] who reported that a carbohydrate/protein drink, which was rich in BCAAs, did not have an effect on muscle soreness, muscle performance or creatine kinase levels following eccentric quadriceps contractions. Our data are also in agreement with Stock et al. [16] who reported that a leucine/carbohydrate beverage provided to volunteers prior to and after 6 sets of heavy back squats did not affect serum creatine kinase, serum lactate dehydrogenase, or muscle soreness at 24 h, 48 h, or 72 h following exercise. Additionally, others have reported that L-leucine administration does not differentially alter serum myoglobin following depth jumps or eccentric leg presses [22]; a finding which is also in agreement with our current data. Therefore, we again contend that BCAA provision likely does not prevent muscle fibers from being damaged during and/or following a training stimulus.

### BCAA-CHO supplementation reduced exercise-induced increases in monocyte differentials versus CHO supplementation

Interestingly, BCAA-CHO supplementation prevented the rise in monocyte percentages compared to the CHO group, although monocyte counts were not altered between or within groups. While aerobic and resistance exercise have been reported to transiently increase leukocyte number and white blood cell differentials [31, 32], levels typically return to baseline within hours of endurance [33] and resistance exercise [32]. Vigorous physical activity has also been shown to induce neutrophil proliferation [34, 35], and intense cycling bouts have been shown to increase neutrophilia and alter neutrophil function [36]. However, in the current study, there were no differences in total WBC or neutrophil counts between or within groups, and this was likely due to the short intermittent duration of the employed weightlifting protocol and/or the 24–48 h post-lifting sampling time points.

Notwithstanding, exercise-induced increases in monocyte percentages can lead to their differentiating into macrophages which, like neutrophils, can infiltrate damaged skeletal muscle following eccentric contractions and potentiate the inflammatory response. In this regard, Malm et al. [37] reported that circulating monocyte numbers and percentages robustly increase up to 7 days following a muscle-damaging eccentric protocol; an effect which paralleled the appearance of macrophages in skeletal muscle tissue. While we are unaware of studies that have examined the ability of BCAA supplementation to reduce post-exercise monocyte proliferation and skeletal muscle macrophage infiltration, it has been recently posited that BCAAs can be transaminated to glutamate in order to increase glutamine synthesis which, in turn, can be consumed by macrophages as a fuel source to propagate inflammation [38]. Notwithstanding, our findings that BCAAs are able to prevent the elevation in monocyte percentages over three days of rigorous weightlifting may suggest that BCAAs may possess an anti-inflammatory properties and should be further researched.

## Conclusions

Our study is not without limitations. First, and as described earlier, while these supplements were not standardized to CHO or Calorie content, our intent was to compare these two supplements in a practical or real-world setting. In this regard, our results may have differed had we had participants consume isocaloric amounts of each supplement. Notwithstanding, we posit that our administration of supplements ascribed to real-world application whereby participants (in free-living environment) would have consumed either the BCAA-CHO or CHO supplement. Second, the training protocol was very brief and does lack a certain degree of external validity (i.e., not many practitioners or athletes would engage in three consecutive days of heavy squats). However, we contend that this study is similar to other laboratory studies which have used comparable weightlifting protocols to elicit muscle damage over short periods of time. Another limitation to this study is that it lacks various mechanistic features; specifically, skeletal muscle biopsies were not obtained and analyzed for infiltrating inflammatory cells and/or histological signs of muscle damage. Thus, we contend that future research needs to employ such mechanistic protocols over longer and more practical training interventions (i.e., ‘overreaching’ paradigms) in order to better determine whether BCAA or BCAA-CHO supplementation can improve markers of muscle damage. Finally, it should be noted that there was not a non-supplemented control condition. Alternatively stated, it is possible that supplement conditions may have reduced the decrements in strength and markers similarly compared to a non-supplement group, but the current study design precludes us from reporting such data. Notwithstanding and despite these limitations, we report that BCAA-CHO supplementation did not reduce decrements in lower body strength or improve select markers of muscle damage/soreness compared to CHO supplementation over three consecutive days of intense lower-body training. As with previous studies reporting similar findings [15, 16, 22], we contend this is due to exercise-induced muscle damage occurring independent of BCAA or CHO provision.

## Abbreviations

1RM, one-repetition maximum; BCAA, branched chain amino acids; CHO, carbohydrate; EMG, electromyography; MVIC, maximum voluntary isometric contraction; USG, urine specific gravity; VAS, visual analog scale; WBC, white blood cell count
